# 利用高通量转录组测序对肺鳞癌基因型的

**DOI:** 10.3779/j.issn.1009-3419.2017.11.01

**Published:** 2017-11-20

**Authors:** 英 雄, 明珍 李, 攀攀 张, 理意 张, 跃 杨

**Affiliations:** 1 100142 北京，北京肿瘤医院暨北京市肿瘤防治研究所恶性肿瘤发病机制及转化研究教育部重点实验室胸外科二病区 Department of Thoracic Surgery Ⅱ, Beijing Cancer Hospital, Key Laboratory of Carcinogenesis and Translational Research(Ministry of Education/Beijing), Peking University Cancer Hospital and Institute, Beijing 100142, China; 2 100089 北京，北京市理化分析测试中心 Beijing Center for Physical and Chemical Analysis, Beijing 100089, China

**Keywords:** 肺肿瘤, 高通量测序, SPRR家族, Lung neoplasms, Transcriptome sequence, SPRR family

## Abstract

**背景与目的:**

目前尚无有效的靶向药物用于肺鳞癌的临床治疗，在肺腺癌中有效的靶向药物却无法让鳞癌患者获益，而研究者对肺鳞癌的靶点知之甚少。本研究重点筛选并鉴定肺鳞癌的特异关键基因，为肺鳞癌的治疗提供新的靶点。

**方法:**

转录组测序检测5例肺鳞癌患者的配对肺癌组织及正常肺组织样本，使用生物信息学分析筛选两组之间的差异编码基因，进而应用实时定量PCR验证关键差异基因在肺癌细胞系的表达情况。

**结果:**

转录组测序结果显示，与正常肺组织相比，肺鳞癌组织上调的差异基因数目为534条。位于前十位肺癌组织高表达的基因包括*GAGE12J*、*SPRR3*、*PRAME*、*SPRR1A*、*SPRR2E*、*MAGEA3*、*SPRR1B*、*IL36G*、*TMPRSS11D*和*SPRR2D*。进而于不同生物特征的肺癌细胞系H520、GLC82、A549、H1299及PC9验证SPRR家族表达状态，我们发现在淋巴结转移细胞系H1299处于高表达水平。

**结论:**

高通量转录组测序筛选到肺鳞癌异常高表达基因SPRR家族，此家族与肺癌淋巴结转移相关，为肺癌的靶向治疗提供了新的思路。

## Abstract (en)

**Background and objective:**

Recently, there is no effective targeted drug for to the lung squamous carcinoma, the targeted drug for the adenocarcinoma does not benefit for the patients with squamous lung cancer, and researchers have known less targets for this type of patients. The main study herein is to screen and identify the specific key genes for the lung squamous carcinoma that will provide potential novel targets to describe pathogenesis of lung cancer.

**Methods:**

Transcript sequence was used to detect the five pairs lung squamous carcinoma and normal lung tissues and bioinformatics was performed to investigate the differential coding genes between them and quantitative PCR was done to validate the key genes expression in lung cancer cell lines.

**Results:**

The results of transcriptome sequence showed that 534 genes were up-regulated in the tumor tissues compared with the corresponding normal tissues. The top ten increased genes in the tumors were *GAGE12J*, *SPRR3*, *PRAME*, *SPRR1A*, *SPRR2E*, *MAGEA3*, *SPRR1B*, *IL36G* and *TMPRSS11D*. Next, we identified expression of SPRR family in lung cancer cell lines H520, GLC82, A549, H1299 and PC9. And we found higher expression of SPRR family genes in H1299 cells which metastasized to the lymph node.

**Conclusion:**

SPRR family genes are identified to be highly expressed in the lung squamous carcinoma by high throughput transcript sequencing. This SPRR family genes are associated with lymph node metastasis that provide novel idea for lung cancer therapy.

目前肺癌的发病率和死亡率在全球范围内位于前列，对公众健康造成极大危害。肺癌的死亡人数在过去的三十年上升了465%，约增长了5倍，已经成为我们国家恶性肿瘤死亡原因中第一位。所以现在人们把肺癌称为我们国家癌症领域的“第一杀手”。其中非小细胞肺癌（non-small cell lung cancer, NSCLC）的发病率占到了85%，主要包括腺癌、鳞癌以及大细胞癌，其中肺鳞癌是最常见的病理类型之一，占NSCLC的30%-40%^[1, 2]^。肺鳞癌又称肺鳞状上皮细胞癌，以中央型肺癌多见，并有胸管腔内生长的倾向，早期常引发支气管狭窄或阻塞性肺炎^[2]^。

目前分子靶向治疗的出现彻底改变了癌症治疗格局。在肺腺癌中，分子靶向治疗获得了较为广泛的应用，但是在肺鳞状细胞癌患者的治疗中还没有实现。高通量二代测序作为一种新的实验技术，能够为我们提供详细、特异的信息，最重要的是它的正确率和高特异性^[3, 4]^。所以从现实意义的角度出发，本研究的目的在于利用高通量二代测序技术，通过查找正常组织与肿瘤组织在基因表达上的差异，为实现肺鳞癌的分子靶向治疗提供基础。因此，我们针对5对肺癌鳞癌患者的肿瘤组织和正常肺组织进行高通量转录组测序，发现差异表达基因，并且对其差异基因SPRR进行肺癌细胞系的验证，有望为肺鳞癌靶向治疗提供新的靶点。

## 1 资料与方法

### 1.1 临床资料

收集5例经病理诊断为肺鳞癌患者的肿瘤组织及正常肺组织的新鲜标本，年龄45岁-65岁。标本收集后分立即置于液氮中速冻，保存于-80 ℃冰箱中，用于实验检测。肺癌细胞系H520、GLC82、A549、H1299及PC9使用1640培养基、37 ℃的CO_2_温箱培养。

### 1.2 总RNA提取

取等量肺癌癌组织及正常肺组织，液氮中磨碎；加入TRIZOL Reagent，室温孵育5 min；加入200 μL氯仿剧烈振荡15 s，室温孵育10 min；4 ℃，12, 000 rpm，离心15 min。吸取上层含有RNA的水相入新管，加500 μL异丙醇沉淀RNA，室温孵育10 min；4 ℃，12, 000 rpm，离心15 min。弃上清，加1 mL的75%乙醇洗涤RNA沉淀，4 ℃，7, 500 rpm，离心5 min，用20 pL DEPC水溶解RNA。用紫外分光光度计测定RNA的含量，-80 ℃冰箱保存。

### 1.3 转录组文库制备及上机测序

利用Oligo dT微珠富集纯化mRNA并进行片段化处理，在3末端加碱基A、连接测序接头，割胶，纯化并回收200 bp-500 bp之间的cDNA片段。PCR扩增后完成测序文库的制备。对测序文库进行质量控制，构件好的文库用Illumina Hiseq2500进行测序，以fastq格式输出。

### 1.4 生物信息分析

将转录组测序样本，分别用TopHat回贴人基因组，其样本比对率需达到90%以上。用Cufflinks分别重新构建转录本，对转录组进行鉴定，合并转录本，得到一个统一的转录本集合。对转录本进行鉴定质量的评估后，最后采用cuffdiff鉴定正常组织样本组和相对应的肺癌组织样本组之间的差异编码基因。

### 1.5 实时定量PCR

使用TRIzol提取肺癌细胞系总RNA，2 μg总RNA逆转成cDNA，使用SYBR Green PCR试剂盒，罗氏480仪器检测差异基因的表达。反应条件，95 ℃ 5 min；95 ℃ 15 s，60 ℃ 30 s，40个循环；72 ℃ 5 min。β-actin作为对照，计算公式：2^-ΔCt^，ΔCt=Ct_目的基因_-Ct_对照_。

## 2 结果

### 2.1 肺鳞癌转录组概况

利用高通量转录组测序技术，检测5对肺癌组织及正常肺组织的标本，利用生物信息学分析筛选差异基因。用cuffdiff鉴定正常组织样本组和相对应的肺癌组织样本组之间的差异基因，发现按照cuffdiff的标准（上下调2倍；*p*-value≤0.05；*q*-value≤0.05），鉴定出来40个差异基因。不考虑q-value之后，差异基因数目明显上升，为534个（*q*-value是基于*p*-value的再统计，*p*-value是样本的一个基本检验几率）。

### 2.2 筛选肺鳞癌组织高表达的前10个基因

基于转录组测序检测5对配对肺癌组织及正常肺组织的标本，重点关注于肺癌肿瘤组织高表达的前10个关键基因（表 1）。正常肺组织测序的FPKM平均值为0.222, 4，肺癌肿瘤组织FPKM平均值为233.4，则肿瘤组织前10位差异基因的表达量是正常组织的1, 049倍。

**1:** 肺鳞癌组织中高表达的前10个基因 The top ten genes highly expressed in the lung squamous carcinoma

Genes	Normal tissues FPKM	Tumor tissues FPKM	Log2 (Fold change)	*P*
*GAGE12J*	0.018, 025, 3	54.519	11.562, 5	0.006, 067, 27
*SPRR3*	0.198, 758	307.336	10.594, 6	9.24E-06
*PRAME*	0.008, 184, 37	11.329	10.434, 9	0.039, 201, 7
*SPRR1A*	0.515, 89	575.838	10.124, 4	0.010, 568, 3
*SPRR2E*	0.125, 185	132.156	10.044	5.65E-07
*MAGEA3*	0.006, 615, 16	6.476, 35	9.935, 19	0.008, 148, 43
*SPRR1B*	0.511, 568	493.149	9.912, 88	8.55E-07
*IL36G*	0.035, 212, 1	33.301, 5	9.885, 3	0.025, 304, 8
*TMPRSS11D*	0.005, 171, 98	4.680, 64	9.821, 78	0.000, 461, 89
*SPRR2D*	0.799, 756	715.034	9.804, 24	6.16E-06

在这10个基因中，*SPRR1A*、*SPRR1B*、*SPRR2D*、*SPRR2E*和*SPRR3*为同一家族成员，其FPKM值在正常肺组织与肿瘤组织中分别为0.430, 2和444.72，二者相差1, 033倍。其中又以SPRR3在肺肿瘤组织与正常组织之间的差异最大，为1, 546倍（表 2）。

**2:** SPRRs家族基因在正常肺组织与肺癌肿瘤组织中的表达 Expression of SPRRs family in the normal tissues and lung cancer tissues

Genes	Normal tissues FPKM	Tumor tissues FPKM	Fold change
*SPRR3*	0.198, 758	307.336	1, 546
*SPRR1A*	0.515, 89	575.838	1, 116
*SPRR2E*	0.125, 185	132.156	1, 055
*SPRR1B*	0.511, 568	493.149	963
*SPRR2D*	0.799, 756	715.034	894

### 2.3 肺癌细胞系中SPRR家族基因的表达情况

采用荧光定量PCR的方法检测了前10位差异基因在肺癌细胞系中的表达情况，见图 1。H520为肺鳞癌细胞，GLC82、A549、H1299、PC9为肺腺癌细胞。其中H1299为淋巴结转移的肺癌细胞系，PC9是表皮生长因子受体酪氨酸激酶抑制剂（epithelial growth factor receptor tyrosine kinase inhibitors, EGFR-TKIs）敏感的肺癌细胞系。从图 1中可以看出SPRR1A、SPRR1B、SPRR2D、SPRR2E和SPRR3在肺癌细胞中广泛表达，此5类亚型于淋巴结转移的H1299细胞中均有较高表达，均高于在未转移的肺腺癌细胞系A549的表达水平。

**1:**
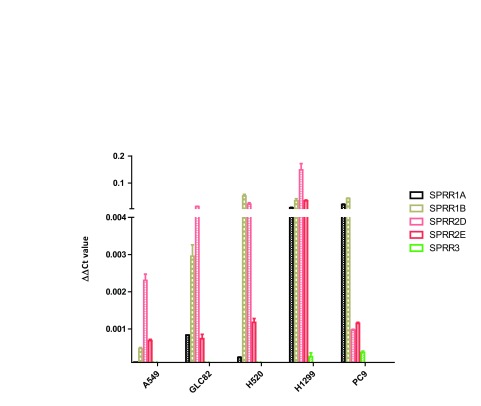
柱状图显示SPRRs家族基因包括*SPRR1A*、*SPRR1B*、*SPRR2D*、*SPRR2E*和*SPRR3*在肺癌细胞系中的表达情况（2^-ΔCt^ value）。 The histogram shows expression of SPRRs family genes including *SPRR1A*, *SPRR1B*, *SPRR2D*, *SPRR2E* and *SPRR3* in the lung cancer cells lines (2^-ΔCt^ value).

## 3 讨论

近十年来，基于NSCLC驱动基因的个体化靶向诊治研究拉开了帷幕，并取得了革命性进展而应用于临床，例如*EGFR*突变基础上的TKIs，基于*EML4-ALK*融合基因的抑制剂等主要应用于肺腺癌^[5]^。然而，针对肺鳞癌的靶向诊治研究十分有限。此外，随着新一代高通量测序技术的快速发展，转录组测序已成为基因表达和转录组分析新的重要手段。转录组测序能够快速并全面的获取组织中几乎所有转录本RNA的序列信息，是目前常用的高通量分析手段，已成为肿瘤研究的重要技术支撑。

本研究正是利用转录组测序技术检测了5对肺癌组织及正常肺组织的基因型的改变，并利用生物信息学分析筛选出与肿瘤密切相关的排在前10位的基因，包括*SPRR1A*、*SPRR1B*、*SPRR2D*、*SPRR2E*、*SPRR3*、*PRAME*、*MAGEA3*、*IL36G*、*GAGE12J*、*TMPRSS11D*。其中，位于差异基因前5位的*SPRR1A*、*SPRR1B*、*SPRR2D*、*SPRR2E*和*SPRR3*基因同属于SPRRs家族，编码一类富含脯氨酸的蛋白^[6]^，而有研究表明这些基因亚型在表达上有一定的偏向性。*SPRR1A*和*SPRR1B*基因表达与口腔上皮细胞的屏障作用相关^[7]^，*SPRR2*基因亚型在消化道疾病以及皮肤病的炎症反应中表达水平有所增加^[8, 9]^。

*IL36G*基因在组织发生损伤与修复过程中表达增高，可见与炎症和免疫反应密切相关^[10, 11]^。也有报道显示*IL36G*基因与皮肤病的炎性反应相关^[12]^。据报道炎症反应能够促进肿瘤的发生，并与肿瘤发生转移密切相关。而本研究结果显示SPRRs家族及IL36G在癌组织高表达的情况可能参与了炎症反应且具有促进肺癌发生的作用。*GAGE12J*基因在胎儿和肿瘤组织中表达，是肿瘤/睾丸抗原家族成员之一。有报道^[13, 14]^发现其在脑膜瘤和神经鞘瘤中可与其他基因相互作用。TMPRSS11D属于Ⅱ型跨膜丝氨酸蛋白酶亚家族成员，在宫颈鳞癌和食管鳞癌的形成过程中缺失^[15]^。PRAME是肿瘤/睾丸抗原的一种，可作为潜在的免疫治疗的靶点，其表达状态与上皮性卵巢癌的启动子低甲基化有关^[16]^。MAGEA3同样也属于肿瘤/睾丸抗原的一种，ORIS与其启动子结合而调控肺癌的转录活性^[17]^。

我们进一步在肺癌细胞系中检测差异基因SPRRs家族的表达状态发现，在淋巴结转移的细胞系H1299中，*SPRR1A*、*SPRR1B*、*SPRR2D*、*SPRR2E*和*SPRR3*均呈高表达状态。前期的研究发现SPRRs家族基因在胆管上皮细胞中能够增加对损伤的抵抗，并与EMT密切相关^[18]^。另有研究^[19]^表明长链非编码RNA MALAT-1通过调控SPRRs家族基因而影响了舌鳞状细胞癌的远端转移。而我们的研究亦发现此家族基因在局部转移的细胞中高表达，提示有可能在肺癌细胞发生转移过程中发挥着重要作用。

目前随着临床研究的深入，证明了个体化的肿瘤治疗相比传统的方法更加安全有效。而本研究利用转录组测序技术，通过查找正常肺组织与肺癌肿瘤组织在基因表达上的差异，为实现肺鳞癌的分子靶向治疗以及寻找更多的分子治疗靶点提供了可行性。
